# Shikonin-Loaded Nanoparticles Attenuate Particulate Matter-Induced Skin Injury by Inhibiting Oxidative Stress and Inflammation

**DOI:** 10.3390/antiox14111301

**Published:** 2025-10-29

**Authors:** Feifei Huang, Qinghua Tang, Ke Wang, Lingmei Zhou, Ruiwei Liao, Zhuoya Wang, Yan Li, Lin Zhou, Ming Li

**Affiliations:** 1 School of Basic Medical Sciences, Guangdong Pharmaceutical University, Guangzhou Higher Education Mega Centre, No. 280 Waihuan East Road, Panyu District, Guangzhou 510006, China; 2112340260@stu.gdpu.edu.cn (F.H.); 2112440144@stu.gdpu.edu.cn (Q.T.); 2112340229@stu.gdpu.edu.cn (L.Z.); wangzhuoya@gdpu.edu.cn (Z.W.); 2 School of Life Science and Biopharmaceutics, Guangdong Pharmaceutical University, Guangzhou Higher Education Mega Centre, No. 280 Waihuan East Road, Panyu District, Guangzhou 510006, China; 2112348092@stu.gdpu.edu.cn; 3 School of Basic Medical Sciences, Guangzhou University of Chinese Medicine, Guangzhou Higher Education Mega Centre, No. 232 East Waihuan Road, Panyu District, Guangzhou 510415, China; liaoruiwei@stu.gzucm.edu.cn (R.L.); liy506@gzucm.edu.cn (Y.L.)

**Keywords:** shikonin, nanoparticles, fine particulate matter, oxidative stress, inflammation

## Abstract

Exposure to fine particulate matter (PM2.5) poses a major threat to skin health, yet effective prevention strategies remain limited. Shikonin, a naphthoquinone derived from Lithospermum erythrorhizon, exhibits potent antioxidant and anti-inflammatory activities. However, its therapeutic application is limited by low bioavailability. To address this limitation, we developed shikonin-loaded nanoparticles (SH-NPs) using an emulsion solvent evaporation method and characterized their physicochemical properties. The protective effects of SH-NPs against PM2.5-induced skin damage were evaluated in a mouse model. The SH-NPs exhibited favorable characteristics, including a mean particle size of 209.03 ± 2.45 nm, a PDI of 0.064 ± 0.03, and a zeta potential of –17.69 ± 2.06 mV. The encapsulation efficiency is 88% and the drug loading capacity is 5.5%, respectively. In vitro, SH-NPs significantly enhanced cellular uptake in HaCaT cells. In vivo, treatment with SH-NPs significantly improved skin structural disorders, epidermal thickening, and collagen fiber reduction, while downregulating the expression of MMP-2 and MMP-9. Furthermore, SH-NPs increased the expression of SOD1 and SOD2, reduced MDA levels, and decreased the expression of TNF-α, IL-1β, and NO. In conclusion, SH-NPs attenuated PM2.5-induced skin toxicity via enhanced antioxidant, anti-inflammatory, and anti-degradation mechanisms, offering a novel strategy to boost shikonin bioavailability and prevent PM2.5-related skin damage.

## 1. Introduction

Ambient air pollution is a serious public health hazard in modern industrialized societies. The World Health Organization (WHO) reported that 4.2 million people die annually from exposure to polluted air [1]. Among air pollutants, PM2.5 is recognized as a major concern and has been designated as a key regulated pollutant under the National Ambient Air Quality Standards (NAAQS) in many countries [2]. PM2.5 consists of various chemical substances of different sizes and shapes, including harmful inorganic metals, carbon compounds, polycyclic aromatic hydrocarbons (PAHs), and volatile organic compounds (VOCs), with PAHs and VOCs constituting the largest proportion of its components [3]. Owing to its small particle size (an aerodynamic diameter ≤ 2.5 µm), PM2.5 can be deposited in the distant lung, causing respiratory system damage, including aggravation of asthma, decreased lung function, increased coughing, or difficulty breathing [4,5]. Researchers also found that PM2.5 can penetrate the alveoli and enter the bloodstream, leading to multiple systemic disorders [6]. The skin serves as a direct interface between the body and the external environment, which may serve as the primary protective interface against environmental stressors such as ultraviolet radiation, ozone, and PM2.5 [7]. Consequently, physical or physiological adverse effects in the epidermis, dermis, and deeper subcutaneous layers of the skin are the earliest response against changes in the surrounding environment [3]. A cross-sectional study by Chen et al. proved the odds of eczema increased with greater PM2.5 concentration in an adult American cohort [8]. And another research has also established a correlation between elevated exposure to air pollutants and an increased incidence of late-onset seborrheic dermatitis [9]. A birth cohort study executed by Lin et al. demonstrated that prenatal and postnatal exposure to PM2.5 is associated with the subsequent development of atopic dermatitis (AD) [10]. Furthermore, Wu et al. reported that each 10 μg/m^3^ increase in PM2.5 concentration was associated with a rise in outpatient visits for dermatitis [11]. A study reported that PM2.5 damages skin cells by inducing oxidative stress, subcellular organelle dysfunction, and apoptosis [12]. Bharrhan et al. highlighted that PM2.5 activated NF-κB, followed by translocation into the nucleus, and induction of inflammatory genes such as IL1α, IL-1β, IL-6, and IL-8 [13]. Kammeyer and Xue also proved that PM2.5 enhances reactive oxygen species (ROS) generation and upregulates matrix metalloproteinase (MMP) expression, which accelerates the degradation of collagen and elastin, ultimately impairing skin structural homeostasis and functional integrity [14,15]. These collective findings suggest that PM2.5 exposure may exacerbate common dermatological diseases through inducing oxidative stress and inflammatory responses. However, therapeutic research on the cutaneous toxicity of PM2.5 is remarkably sparse.

Therapeutic agents that concurrently suppress ROS overproduction and inhibit inflammatory cytokine release hold considerable promise for mitigating PM2.5-induced skin injury. Shikonin, a natural naphthoquinone found in Lithospermum erythrorhizon, has attracted extensive research interest owing to its potent pharmacological activities. It is renowned for its wide spectrum of biological effects, most notably its significant anti-inflammatory, antioxidant, and antimicrobial properties, which have established it as a promising candidate for therapeutic development [16,17]. Yang et al. discovered that shikonin attenuates ISO-induced cardiomyopathy by reducing fibrosis, inflammation, apoptosis, and endoplasmic reticulum stress [18]. Peng et al. found that shikonin alleviates lipopolysaccharide (LPS)-induced apoptosis, oxidative stress, and inflammatory responses in renal tubular epithelial cells by modulating the NOX4/PTEN/AKT pathway [19]. Shikonin is also widely applied in the research of the prevention and treatment of skin diseases. Yu et al. proved shikonin could inhibit keratinocyte proliferation and induce cellular apoptosis, thereby ameliorating psoriasis through a JAK/STAT3-dependent pathway [20]. Tong et al. demonstrated that shikonin treatment effectively enhanced PC12 cell viability, reduced malondialdehyde (MDA) content and ROS levels, and stabilized mitochondrial membrane potential [21]. Furthermore, shikonin could increase the activities of key antioxidant enzymes, including catalase (CAT), superoxide dismutase (SOD), and glutathione peroxidase (GPx), thereby significantly alleviating oxidative damage [22]. However, the water solubility of shikonin is relatively low, resulting in poor bioavailability and limited potential for clinical application.

Nanoparticle-based drug delivery systems offer a promising strategy to enhance the dermal delivery efficiency of hydrophobic active compounds [23]. Among these, poly (lactic-co-glycolic acid) (PLGA), a biodegradable polymer approved by the U.S. Food and Drug Administration (FDA), exhibits excellent biocompatibility, controllable release properties, and high drug-loading capacity. These systems are particularly beneficial for insoluble drugs, as they can significantly enhance drug solubility and have been widely explored for dermal applications. Park et al. demonstrated that curcumin-loaded PLGA-based discoidal polymeric particles effectively targeted the lungs and significantly alleviated airway inflammation in a murine asthma model. The treatment exhibited superior efficacy compared to free curcumin, without inducing notable systemic toxicity [24]. Similar results were obtained by Bhatt et al. in the resveratrol-loaded PLGA nanoparticles [25]. This integrated system can prolong drug retention and enhance skin penetration through a “skin reservoir effect”, which ensures efficient solubilization, protection, and sustained delivery of insoluble therapeutics to the skin [26,27]. Kshirsagar et al. utilized PLGA nanoparticles to encapsulate tazarotene, demonstrating that this nano-system enables sustained drug release, enhances follicular delivery, and maintains drug bioactivation capability [28]. These findings provide a solid foundation for utilizing PLGA-based carriers to improve the delivery efficiency of shikonin to the skin.

Therefore, this study aims to develop shikonin-loaded PLGA nanoparticles (SH-NPs) to overcome the inherent physicochemical limitations of shikonin and assess its potential protective efficacy against PM2.5-induced skin damage. A comprehensive characterization of SH-NPs, including particle size, zeta potential, encapsulation efficiency, and drug release profile, will be conducted. Furthermore, using a PM2.5-induced mouse skin injury model, the therapeutic effects of SH-NPs will be systematically evaluated at macroscopic, histopathological, and molecular levels, with emphasis on their ability to alleviate oxidative stress, suppress inflammatory responses, and maintain extracellular matrix homeostasis. This study is expected to provide not only a novel nanotherapeutic strategy for the prevention and treatment of PM2.5-related skin disorders but also a technical reference and theoretical basis for efficient dermal delivery of natural bioactive compounds.

## 2. Materials and Methods

### 2.1. Preparation of Shikonin-Loaded PLGA Nanoparticle

The main materials and their suppliers used in the process of synthesizing nanoparticles were as follows: shikonin (CAS No. 517-89-5, purity >98%) from Beyotime Biotechnology (Shanghai, China); PLGA (LA/GA = 50:50, M_n_ ≈ 15,000) from Chuangsai Biotech (Guangzhou, China); and acetone along with polyvinyl alcohol (PVA) from Macklin (Shanghai, China).

SH-NPs were constructed according to the methodology reported by our research team previously, with some modifications [29]. The constructions began with the formulation of organic and aqueous phases. Briefly, 5 mg of shikonin and 10 mg of PLGA were completely dissolved in 5 mL of acetone to form a homogeneous organic phase. Simultaneously, an aqueous phase was prepared by dissolving polyvinyl alcohol PVA in distilled water to obtain a 1% (*w*/*v*) aqueous solution. The organic phase was then gradually added to the PVA solution under constant stirring, followed by sonication at 200 W for 5 min to form the final emulsion. Then, the emulsions were stirred for 24 h to volatilize the organic phase. The resulting suspension was filtered through a 0.45 μm membrane to collect the SH-NPs. Finally, the obtained SH-NPs were lyophilized using a Labconco FreeZone Triad Freeze Dryer (HFD-1, Guangzhou, China) to obtain a dry powder for subsequent experiments (Figure S1). The process includes the following main modifications: the mass ratio of shikonin to PLGA was optimized to enhance drug loading capacity; based on preliminary data showing higher drug loading after longer sonication (5 min: 3.5% vs. 2 min: 2.3%), the ultrasonication time was set to 5 min, thereby also promoting the formation of smaller and more homogeneous particles; Furthermore, the stirring duration was extended from 4 h to 24 h to ensure the complete evaporation of the organic solvent. The lyophilized program is as follows: The lyophilization cycle was developed and performed as follows: The samples were loaded onto the shelves pre-cooled to +5 °C. The freezing phase consisted of cooling to −45 °C at 1 °C/min and holding for 3 h. Primary drying was conducted at a shelf temperature of −25 °Cand a chamber pressure of 100 μHg (≈133 Pa) for 40 h. Secondary drying was initiated by raising the shelf temperature to +30 °C over 2 h and maintaining it for 12 h at the same chamber pressure. The vials were sealed under a nitrogen atmosphere while still on the shelf.

### 2.2. Characterization of Shikonin-Loaded PLGA Nanoparticles

#### 2.2.1. Particle Size and Zeta Potential

The average hydrodynamic diameter, polydispersity index (PDI), and Zeta potential of SH-NPs were determined using a NanoBrook Omni particle size and zeta potential analyzer (Brookhaven Instruments Corporation, Nashua, NH, USA). Before measurement, the nanoparticle suspension was diluted with deionized water at a 1:10 (*v*/*v*) ratio and ultrasonicated for 5 min to ensure homogeneity. All measurements were performed at 25 °C with an equilibration time of 90 s. The scattering angle was set at 90 degrees for particle size analysis. Particle size distribution and PDI values are expressed as mean ± standard deviation (n = 3), with each measurement consisting of 3 consecutive runs. Zeta potential of SH-NPs was measured under the same conditions at a constant temperature of 25 °C, with the instrument’s voltage and frequency in automatic mode. Results represent the mean ± standard deviation of three independent measurements (n = 3).

#### 2.2.2. Morphology

The morphology of SH-NPs was characterized by transmission electron microscopy (TEM) (Hitachi, Tokyo, Japan) following the method described by Li et al. [30]. A droplet of the SH-NPs suspension was deposited onto a copper grid and allowed to settle for 1 min, after which excess liquid was carefully removed using filter paper. Subsequently, the sample was negatively stained with a droplet of 2% phosphotungstic acid (Ron Reagent, Shanghai, China) for 1 min, and the excess stain solution was removed carefully. After air-drying at room temperature for several minutes, the grid was imaged under TEM, and representative micrographs were acquired for analysis.

#### 2.2.3. Drug-Loading and Encapsulation Efficiency

The drug loading (DL) and encapsulation efficiency (EE) of SH-NPs were determined by an indirect method with UV-visible spectrophotometry. A precisely weighed 1.0 mg sample of lyophilized SH-NPs was dissolved in 1 mL of dichloromethane (Macklin, Shanghai, China). The mixture was sonicated in an ultrasonic bath (Model KQ3200, 40 kHz) for 10 min to ensure complete dissolution. The solution was centrifuged at 12,000 rpm for 10 min, and the resulting residue was redissolved in 10 mL of anhydrous ethanol (Guangzhou Guanghua Technology Co., Ltd., Guangzhou, China) followed by vortexing for 2 min. Before to quantification, a full-wavelength scan was performed using a UV-visible spectrophotometer (Shimadzu UV-2600, Tokyo, Japan), which confirmed the characteristic absorption peak of SH-NPs at 215 nm. The concentration was determined using a pre-established calibration curve (concentration range: 2–12 μg/mL, R^2^ = 0.9992) with anhydrous ethanol as the blank. The DL percentage (% DL) was determined by dividing the weight of the drugs in the SH-NPs by the total weight of the nanoparticles. The EE percentage (% EE) was calculated by dividing the weight of drugs encapsulated in the SH-NPs by the initial weight of the drug used [31,32].

The formula for % DL:

(1) $$\%\;\mathrm{DL}\;=\;\frac{Weight\;of\;drug\;in\;nanoparticles}{Weight\;of\;nanoparticles}\times 100$$

The formula for % EE:

(2) $$\%\;\mathrm{EE}\;=\;\frac{Weight\;of\;drug\;in\;nanoparticles}{Weight\;of\;the\;feeding\;drug}\times 100$$

#### 2.2.4. Evaluation of Cellular Uptake of Nanoparticles

For evaluation of the cellular uptake, a coumarin-6-loaded nanoparticle (C6-NPs) was prepared following the same procedure as that for SH-NPs, except that shikonin in the organic phase was replaced with the fluorescent marker C6 (Beyotime Biotechnology, Shanghai, China).

HaCaT cells (Suyan Biotechnology, Guangzhou, China) were cultured in Dulbecco’s Modified Eagle Medium (DMEM) medium (Procell, Wuhan, China) supplemented with 10% fetal bovine serum (Procell, Wuhan, China) and 1% penicillin-streptomycin (Gibco, Waltham, MA, USA) in an incubator with 5% CO_2_ at 37 °C. Cells were seeded into 6-well plates with a concentration of 150,000 cells per well and cultured for 24 h. The cells were then treated with C6-NPs and incubated for another 24 h at 37 °C. The cells were washed three times with PBS to remove excess C6-NPs that were not taken up by the cells. Then, 4% paraformaldehyde was added to fix the cells, washed three times with PBS, and stained the nuclei with Hoechst 33342 reagent. The uptake of NPs by cells was observed using a fluorescence microscope (Olympus, Tokyo, Japan).

### 2.3. Preparation of PM2.5

PM2.5 (Cat. No. SRM1649b) was purchased from the National Institute of Standards and Technology (NIST, Gaithersburg, MD, USA). It is a commonly used standard reference in PM2.5 toxicological research [33]. Its main components include carbonaceous components (organic carbon and elemental carbon), polycyclic aromatic hydrocarbons (e.g., anthracene and pyrene), metals and trace elements (such as lead, arsenic, cadmium, chromium, and zinc), along with water-soluble ions like nitrates and ammonium salts. The detailed components of this PM2.5 can be found at https://www-s.nist.gov/srmors/view_detail.cfm?srm=1649b, accessed on 19 October 2025. PM2.5 particles were suspended in PBS to obtain a stock concentration of 10 mg/mL. To prevent particle aggregation, the sample was sonicated in an ultrasonic bath for 10 min before each application.

### 2.4. Experimental Animals

Thirty male BALB/c mice, aged 6–8 weeks (body weight 18 ± 2 g), were purchased from the Guangdong Experimental Animal Center and used in the present study. All animal experiments were approved by the Animal Care and Use Professional Committee of Guangdong Pharmaceutical University (License No. GDPULACSPF2022833. Animal Approval Date: 23 May 2025) and strictly followed the National Institutes of Health Guide for the Care and Use of Laboratory Animals. The mice were fed standard laboratory food and water under controlled conditions (12 h:12 h light: dark rhythm, 23 ± 2 °C ambient temperature, 45–55% humidity).

The day before modeling, the dorsal hair of all mice was removed using a non-irritating depilatory cream to expose a skin area of approximately 2 × 2 cm^2^. Then the mice were randomly divided into six groups (n = 5): healthy control group (CON), model control group (MC), 0.5% SH-NP group, 1% SH-NP group, 2% SH-NP group, and dexamethasone group (positive control, China Resources Sanjiu Medical & Pharmaceutical Co., Ltd., Shenzhen, China). SH-NPs were dispersed with PBS concentration and used in the treatment. Animals were randomly assigned to each group and all experimental procedures, including drug administration, outcome measurement, and data analysis, were performed by investigators blinded to group identity.

The mice were treated according to their respective groups: the CON group consisted of healthy mice receiving no exposure to PM2.5, propylene glycol, or other solvents; the MC group was composed of mice treated with PM2.5 suspended in propylene glycol; the dexamethasone group was treated with an appropriate amount of dexamethasone ointment; and the 0.5%, 1%, and 2% SH-NP groups were topically administered 500 μL of the corresponding concentration of SH-NP nanosuspension, respectively. Four hours after topical administration of SH-NPs/dexamethasone, PM2.5 was dispersed in propylene glycol to the concentration of 100 µg/mL, spread on a nonwoven polyethylene pad over a 1 cm^2^ area, which was then affixed to the depilated area of the mice using Tegaderm transparent dressing, gauze patches (3M, Guangzhou, China), and Vetrap bandage (3M, China) for secure fixation. The topical treatment and the PM2.5 contained patch were replaced daily, and the mice were weighed and photographed each day throughout the 7-day intervention period [34].

At the end of the intervention (day 8), all mice were euthanized by CO_2_ asphyxiation. Dorsal skin tissue, along with heart, liver, spleen, lung, and kidney samples, were collected for subsequent experimental analysis.

### 2.5. Histopathological Examination

Following fixation in 4% paraformaldehyde for 24 h, skin tissue samples were processed into 5 μm thick paraffin sections. These sections were then stained with hematoxylin and eosin (H&E) and evaluated by standard light microscopy. Collagen deposition was further assessed using Masson’s trichrome staining. The thickness of the skin and collagen deposition were measured and calculated using ImageJ software (https://fiji.sc/, accessed on 8 July 2025) according to Song et al. [35]. Histological analysis of skin tissue samples from three randomly selected mice per group was independently assessed by two investigators. For each sample, images were acquired from three randomly chosen, non-overlapping fields that exhibited intact dermal and epidermal structures.

### 2.6. Immunohistochemical Analysis

Immunohistochemical analysis was conducted as previously described [35]. Briefly, wound tissue sections were deparaffinized, rehydrated, and boiled in 100 °C citrate antigen retrieval buffer (pH = 6.0) for 5 min. Subsequently, the sections were incubated overnight at 4 °C with primary antibodies against MMP-2 (dilution 1:300; cat. no. GB11130) and MMP-9 (dilution 1:200; cat. no. GB11132), both obtained from Servicebio Biotechnology Co., Ltd. (Wuhan, China). Following primary antibody incubation, the sections were washed three times with PBS and then treated with a horseradish peroxidase (HRP)-conjugated goat anti-rabbit IgG secondary antibody and an avidin–biotin peroxidase complex. Signal detection was performed using 3,3′-diaminobenzidine (DAB) followed by counterstaining with hematoxylin. For each group, an independent assessment was performed by two researchers on three randomly selected skin tissue samples. The quantitative analysis involved measuring the percentage of positively stained cells in three random fields per sample using ImageJ software.

### 2.7. Quantitative Real-Time PCR

The mRNA expression levels of inflammatory factors (TNF-α, IL-6) and oxidative stress-related markers (SOD1, SOD2) in mouse dorsal skin tissue were detected using quantitative real-time polymerase chain reaction (qRT-PCR). Total RNA was extracted from skin samples using an RNA extraction kit (Albatross, Guangzhou, China). The RNA concentration and purity were measured with a Nano-300 instrument (Allsheng, Hangzhou, China). Total RNA was reverse-transcribed into complementary DNA (cDNA) using a commercial reverse transcription kit, according to the manufacturer’s instructions (Albatross, Guangzhou, China). The qPCR reaction was performed using a SYBR Green kit (Albatross, Guangzhou, China) with the following amplification protocol: initial denaturation at 95 °C for 30 s, followed by 40 cycles of denaturation at 95 °C for 10 s, and annealing/extension at 60 °C for 30 s. The relative expression levels of the target genes were calculated using the 2^(−ΔΔCT)^ method. The primer sequences used in this study are listed in Table 1.

### 2.8. Detection of MDA

The concentration of malondialdehyde (MDA) was determined with an MDA assay kit (Beyotime Biotechnology, Shanghai, China). Specifically, a 10% homogenate was prepared from 25 mg of skin tissue in lysis buffer and centrifuged (12,000× *g*, 10 min, 4 °C). The supernatant was collected for protein quantification using a BCA kit (Beyotime Biotechnology, Shanghai, China). As per the manufacturer’s instructions, the 0.37% (*w*/*v*) TBA solution, MDA working solution, and standards were prepared. After the samples were mixed with reagents, they were subjected to incubation at 100 °C for 15 min. Subsequently, the mixtures were cooled and centrifuged (1000× *g*, 10 min). The absorbance of the supernatant was then measured at 532 nm with a microplate reader (Shuichuang Medical Devices Co., Ltd., Beijing, China), and MDA levels were calculated based on the absorbance values. The MDA concentration was calculated and expressed as nanomoles per milligram of protein (nmol/mg protein).

### 2.9. Detection of Nitric Oxide (NO)

The concentration of NO was measured using a commercially available assay kit (Beyotime Biotechnology, Shanghai, China). Summarily, 25 mg of skin tissue was homogenized in lysis buffer to obtain a 10% (*w*/*v*) tissue homogenate. The homogenate was subjected to centrifugation (12,000× *g*, 10 min, 4 °C) to collect the supernatant. Subsequently, 50 μL of the supernatant was incubated with 50 μL of Griess Reagent I and 50 μL of Griess Reagent II. The absorbance was measured at 540 nm using a microplate reader, and the NO concentration was determined based on a standard curve derived from the absorbance values. The nitric oxide level was determined using the concentration (μM).

### 2.10. Biosafety Study

Body weights of the mice were measured daily under fasting conditions from day 1 to day 7 of the modeling period using a laboratory animal scale. On day 8, all animals were euthanized, and major organs, including the heart, liver, spleen, lungs, and kidneys, were collected from each group for subsequent histopathological analysis with standard procedures.

### 2.11. Statistical Analysis

Data were analyzed using GraphPad Prism 9.5.0 software. All quantitative results are reported as the mean ± standard deviation (SD). Statistical significance was assessed by one-way analysis of variance (ANOVA) with Tukey’s test applied for post hoc comparisons. A *p*-value of less than 0.05 was defined as the threshold for statistical significance.

## 3. Results

### 3.1. Characterization of Shikonin-Loaded PLGA Nanoparticles

**Figure 1:**
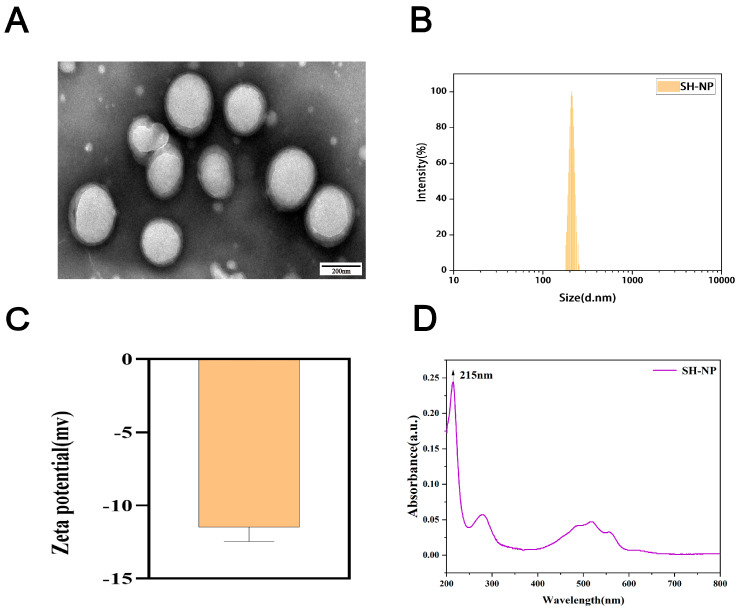
Characterization of shikonin-loaded PLGA nanoparticles. (**A**) TEM image of shikonin-loaded PLGA nanoparticles. (**B**) Particle size distribution of shikonin-loaded PLGA nanoparticles. (**C**) Zeta potential of shikonin-loaded PLGA nanoparticles. (**D**) UV absorption spectrum of shikonin-loaded nanoparticles.

SH-NPs were successfully synthesized and characterized based on their particle size, PDI, and zeta potential. TEM imaging revealed that the SH-NPs possessed a spherical morphology with an average diameter of 161 ± 8.95 nm (Figure 1A). Dynamic light scattering (DLS) measurements indicated a hydrodynamic size of 209.03 ± 2.45 nm and a PDI of 0.064 ± 0.03 (Figure 1B), demonstrating a narrow size distribution. The zeta potential was determined to be −17.90 ± 2.06 mV (Figure 1C), suggesting moderate colloidal stability. UV-Vis spectrophotometric analysis exhibited a characteristic absorption peak at 215 nm (Figure 1D), consistent with the spectral profile of shikonin. Overall, these physicochemical properties confirm that the SH-NPs meet the essential requirements for nanomaterial-based drug delivery systems.

### 3.2. Drug-Loading and Encapsulation Efficiency

Using UV spectrophotometry and the equations in Section 2.2.3, the drug loading capacity and encapsulation efficiency were determined to be 5.5% (Equation (1)) and 88% (Equation (2)), respectively. These values confirm the successful and efficient incorporation of shikonin into the PLGA nanoparticle matrix, which is a crucial prerequisite for achieving the therapeutic effects observed in the subsequent biological experiments.

### 3.3. Assessment of Cellular Uptake

**Figure 2:**
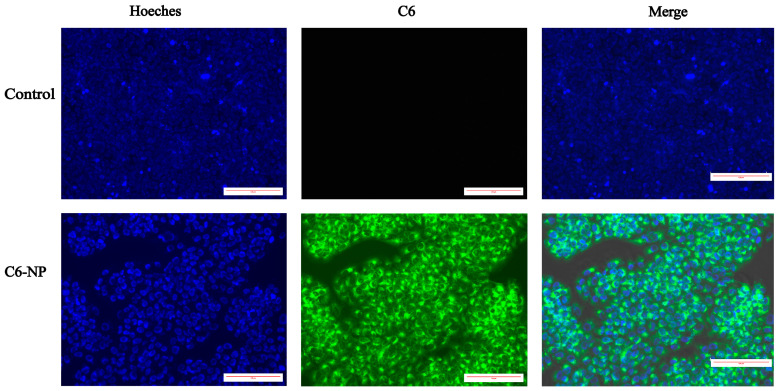
Assessment of cellular uptake. Cellular uptake of C6-labeled nanoparticles in HaCaT cells after 24 h treatment. Blue: DAPI (nuclei); Green: C6-labeled nanoparticles (cytoplasmic distribution).

It is a commonly used method to employ hydrophobic fluorescent compounds as substitutes for hydrophobic drugs to enable visualization and tracking during delivery processes [36,37,38,39]. In the present study, coumarin-6 (C6) was used as a fluorescent surrogate for shikonin and successfully encapsulated into nanoparticles to simulate drug delivery behavior. As shown in Figure 2, qualitative assessment of cellular uptake via fluorescence microscopy revealed intense green fluorescence signals in the cytoplasm of HaCaT cells treated with C6-NPs. In contrast, untreated cells exhibited only blue Hoechst-stained nuclei without any specific fluorescence. These results indicate that the nanoparticles effectively promote the internalization of their cargo into HaCaT cells, thereby providing experimental evidence for enhanced intracellular drug delivery and potential protective efficacy.

### 3.4. Shikonin-Loaded PLGA Nanoparticles Attenuate PM2.5-Induced Skin Injury

**Figure 3:**
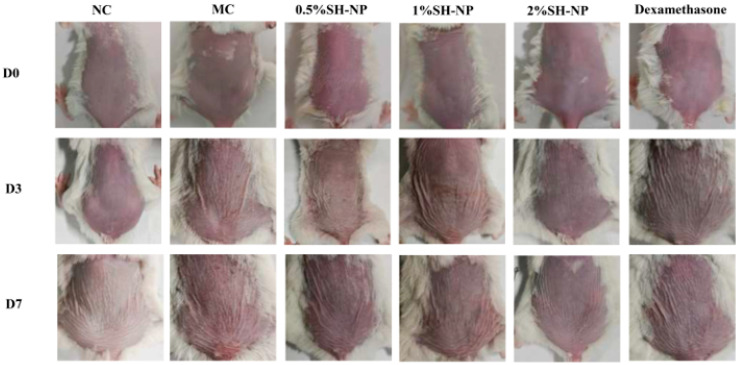
Changes in the dorsal skin of mice. Dorsal skin changes in mice from different groups at days 0, 3, and 7.

The protective efficacy of SH-NPs was evaluated in a murine model of PM2.5 exposure. Figure 3 illustrates the dynamic changes in skin phenotype observed across different experimental groups during modeling and drug administration. The normal control (NC) group exhibited intact skin morphology without pharmacological intervention. In contrast, the model control (MC) group displayed significant pathological alterations, including erythema, skin shrinkage, and mild desquamation, which markedly differed from the NC group, indicating successful model establishment. Compared with the MC group, all SH-NP treatment groups showed varying degrees of improvement. The 0.5% SH-NP group exhibited reduced skin shrinkage and attenuated erythema. The 1% SH-NP group demonstrated more pronounced amelioration in both parameters, and the 2% SH-NP group displayed nearly normal skin morphology with minimal shrinkage and erythema. In the dexamethasone group, skin shrinkage remained largely unimproved, although erythema was partially alleviated. These results indicate that SH-NP intervention effectively ameliorated PM2.5-induced skin damage, characterized by reduced erythema and improved skin shrinkage.

### 3.5. Histopathological Examination of Skin Tissue

**Figure 4:**
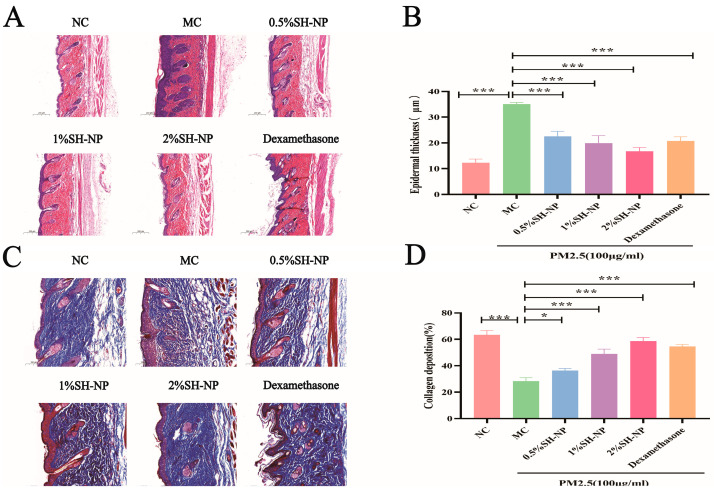
Histopathological examination of skin tissue. (**A**) H&E staining after 7 days of treatment (×200). (**B**) Changes in epidermal thickness of the skin. (**C**) Masson’s staining after 7 days of treatment (×100). (**D**) Quantification of relative collagen area. The data are expressed as mean ± SD (* *p* < 0.05, *** *p* < 0.001, n = 3).

The pathological changes and content of collagen were assessed by HE staining and Masson staining, and the results were shown in Figure 4A and Figure 4C, respectively. The results of HE staining showed skin tissues from the MC group exhibited disrupted architecture, loss of structural integrity, and significant epidermal hyperplasia. In contrast, mice treated with 0.5% SH-NPs, 1% SH-NPs, 2% SH-NPs, and dexamethasone showed dose-dependent improvements in skin histology, including restored tissue organization, enhanced structural integrity, and markedly reduced epidermal thickening. These improvements of epithelial thickness were statistically significant compared to the MC group (*p* < 0.05, as shown in Figure 4B). The skin structure in the MC group was highly disorganized, characterized by fragmented collagen fibers, dispersed fiber bundles, and a complete loss of parallel alignment. This aberrant morphology resulted in a loose, twisted architecture that is closely associated with increased skin wrinkling. Conversely, from the results obtained, it was found that the SH-NPs group reduces the number of wrinkles, increases skin elasticity, as well as more abundant and organized collagen fibers. The collagen deposition in the SH-NPs treatment group was statistically significant relative to the MC group (*p* < 0.05, as shown in Figure 4D).

### 3.6. Immunohistochemical Analysis

**Figure 5:**
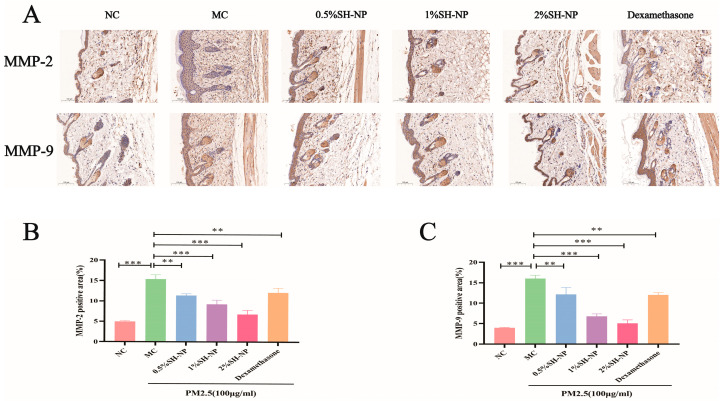
MMP-2 and MMP-9 expression in mouse skin tissue assessed by immunohistochemical Analysis. (**A**) Immunohistochemical staining image of MMP-2 and MMP-9 (×100). (**B**) Quantitative evaluation of the MMP-2 protein in mouse skin. (**C**) Quantitative evaluation of the MMP-9 protein in mouse skin. The data are expressed as mean ± SD (** *p* < 0.01, *** *p* < 0.001, n = 3).

To elucidate the mechanism underlying the protective effects of SH-NPs against PM2.5-induced collagen loss, the expression levels of MMP-2 and MMP-9 in skin tissues were examined with immunohistochemical assay. As demonstrated in Figure 5A–C, significantly higher expression of MMP-2 and MMP-9 was observed in the MC group compared to the NC group. In contrast, administration of SH-NPs at various concentrations led to a notable dose-dependent downregulation in the expression of both proteins.

### 3.7. Shikonin-Loaded PLGA Nanoparticles Alleviate PM2.5-Induced Oxidative Stress

**Figure 6:**
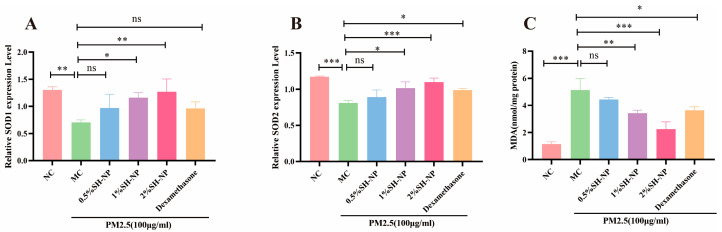
Shikonin-loaded PLGA nanoparticles alleviate PM2.5-induced oxidative stress. (**A**,**B**) Relative mRNA expression of SOD1 and SOD2 in mouse skin after 7 days of treatment. (**C**) Measurement of MDA content in mouse skin after 7 days of treatment. The data are expressed as mean ± SD (* *p* < 0.05, ** *p* < 0.01, *** *p* < 0.001, n = 3).

The expression levels of oxidative stress-related markers SOD1 and SOD2 were detected by qRT-PCR, and the content of MDA was measured using a commercial MDA assay kit. As shown in Figure 6, the MC group exhibited a significant decrease in SOD expression level and a notable increase in MDA content compared to the NC group, with both differences being statistically significant (*p* < 0.05). In contrast, administration of 0.5% SH-NPs, 1% SH-NPs, 2% SH-NPs, and dexamethasone significantly enhanced SOD1 and SOD2 expression and reduced MDA levels in skin tissues, showing statistically significant differences to the MC group (*p* < 0.05). These results indicated that inhibiting oxidative stress is one of the mechanisms by which SH-NPs protect against PM2.5-induced skin injury.

### 3.8. Shikonin-Loaded PLGA Nanoparticles Reduce PM2.5-Induced Inflammatory Responses

**Figure 7:**
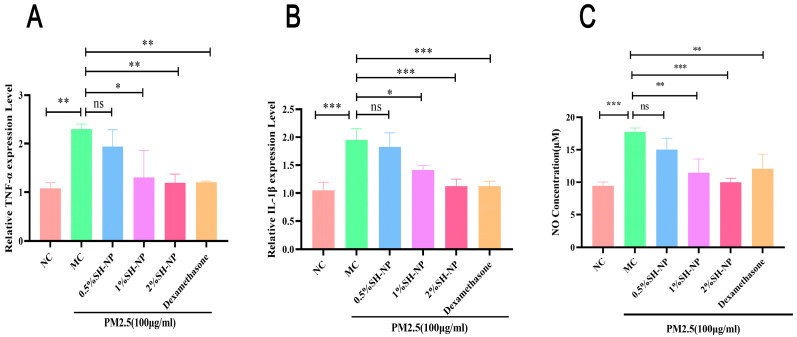
Shikonin-loaded PLGA nanoparticles reduce PM2.5-induced inflammatory responses. (**A**,**B**) Relative mRNA expression of cytokines (TNF-α, IL-1β) in mouse skin after 7 days of treatment. (**C**) NO concentration in mouse skin after 7 days of treatment. The data are expressed as mean ± SD (* *p* < 0.05, ** *p* < 0.01, *** *p* < 0.001, n = 3).

Exposure of skin to environmental pollutants can trigger the upregulation of inflammatory cytokines and associated mediators, resulting in cutaneous damage. As demonstrated in Figure 7, exposure to PM2.5 resulted in a marked upregulation of key pro-inflammatory mediators, including TNF-α, IL-1β, and nitric oxide (NO), in the skin tissues of mice from the MC group. In contrast, treatment with different concentrations of SH-NPs markedly reduced the production of these inflammatory markers, restoring them to levels comparable to those in normal controls. These findings suggest that SH-NPs confer anti-inflammatory protection against PM2.5-induced skin injury by suppressing the expression of pro-inflammatory factors.

### 3.9. Biosafety Evaluation

**Figure 8:**
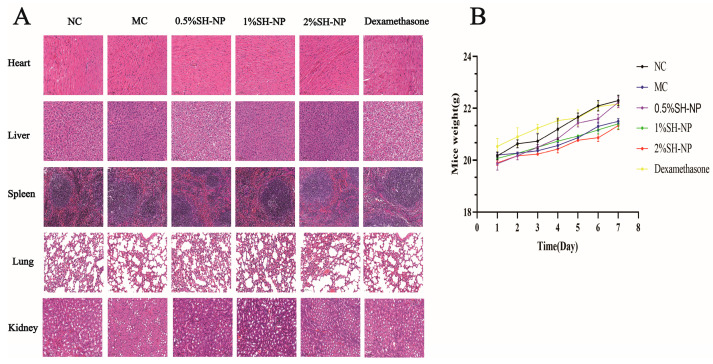
Biosafety evaluation. (**A**) The liver, heart, lungs, spleen, and kidneys were subjected to hematoxylin-eosin (H&E) staining (magnification, ×100). (**B**) Body weight of mice from day 1 to day 7.

To evaluate the biosafety of SH-NPs, a systematic safety assessment was conducted. Histopathological analysis of the heart, liver, spleen, lungs, and kidneys revealed no significant morphological alterations compared with the NC group (Figure 8A). Furthermore, compared with the control group, the mice in the SH-NP groups did not show any significant decrease in weight, and could drink, eat, and move normally, which indicated that the SH-NPs had no obvious toxicity (Figure 8B).

## 4. Discussion

Environmental pollutants, especially PM2.5, have become a globally recognized public health concern due to their harmful effects on skin health. It has been reported by several studies that PM2.5 exposure is associated with various skin diseases, including eczema, psoriasis, accelerated skin aging, and even skin cancer [40]. Consequently, the development of effective protective strategies has become a critical focus. In this study, SH-NPs were developed using an emulsion solvent evaporation method, and their protective effects and underlying mechanisms against PM2.5-induced skin damage were systematically evaluated. Paves the way for the application of SH-NPs as a natural and effective active ingredient in both cosmetic and dermatological fields to mitigate the adverse cutaneous effects of air pollution.

The physicochemical properties of nanoparticles, particularly size and surface charge, are critical determinants of their efficacy and applicability in topical dermatological formulations [41]. Studies have indicated that nanoparticles with a size below 300 nm and a PDI lower than 0.3 can facilitate the delivery of encapsulated agents into deeper skin layers [42,43]. Fu et al. synthesized SH-NPs via the emulsion solvent evaporation method and demonstrated that the SH-NP gel was effective for topical administration in the treatment of psoriasis [44]. In the present study, we successfully synthesized SH-NPs with a suitable particle size, uniform distribution (PDI < 0.3), high encapsulation efficiency, and drug loading capacity via the emulsion solvent evaporation method. Their physicochemical properties meet the fundamental requirements for topical delivery systems [45]. Cell uptake assays further verified the cellular internalization capacity of the formulation. Collectively, the SH-NPs establish the physicochemical foundation for subsequent topical skin application and demonstrate significant potential in mitigating PM2.5-induced skin damage.

PM2.5-induced skin damage can cause a range of characteristic clinical symptoms and pathological changes, significantly compromising skin health and appearance. This study successfully established a mouse model of PM2.5-induced skin damage. Histological and macroscopic evaluations revealed characteristic pathological changes in the model group, including pronounced erythema, hyperkeratosis, and epidermal roughness, and microscopic observation showed that the thickness of the epidermis is significantly increased and the subcutaneous collagen is reduced. These pathological changes, which suggest the skin toxic effects of PM2.5, are consistent with findings from previous studies and epidemiological investigations [34].

Shikomin, the main active ingredient of the traditional Chinese medicinal plant Lithospermum erythrorhizon, has been widely used for decades to treat inflammatory skin diseases. According to Jeong et al., shikonin extract effectively alleviates UVB-induced photoaging [46]. Wang et al. reported that shikonin notably improved dermatitis symptoms and reduced the Psoriasis Area and Severity Index (PASI) score in psoriasis patients [47]. Furthermore, research by Li et al. showed that shikonin also suppresses chemically induced skin carcinogenesis [48]. In this study, SH-NPs were used to protect the skin exposed to PM2.5. The results demonstrated that intervention with SH-NPs significantly alleviated damage caused by PM2.5 exposure, as evidenced by reduced erythema, desquamation of scales, and restoration of skin smoothness and gloss to nearly normal levels. These findings demonstrate the potent reparative efficacy of SH-NPs against PM2.5-mediated skin injury. In summary, the present study and others indicate that shikonin and its agents (such as SH-NPs) confer broad-spectrum protective and therapeutic benefits against diverse skin injuries induced by environmental pollutants, ultraviolet radiation chemical irritants, and immune-mediated dermatological conditions. The mechanisms underlying their efficacy appear to involve multi-factorial pathways rather than reliance on a single pathogenic mechanism.

Collagen fibers are a core structural component of the dermal layer, and their structural integrity plays a crucial role in maintaining skin elasticity and barrier function. Matrix metalloproteinases (MMPs), particularly MMP-2 and MMP-9, are key enzymes responsible for collagen degradation. The dynamic balance between MMP activity and collagen metabolism is essential for the homeostasis of the extracellular matrix [49]. Aberrant upregulation of MMPs can lead to excessive collagen breakdown, disruption of skin architecture, reduced elasticity, and ultimately an accelerated aging process [50]. Using Masson’s staining and IHC assay, our study found that exposure to PM2.5 significantly upregulates the expression of MMP-2 and MMP-9, resulting in disorganized collagen arrangement, increased fiber spacing, and reduced density. This partly explained the mechanisms by which PM2.5 exposure induced skin damage, especially skin shrinkage. Bae et al. also proved PM2.5 could enhance the expression of MMP-1 in a 3-D-skin model [51]. Our results from IHCs demonstrated that treatment with SH-NPs markedly suppressed the expression of MMP-2 and MMP-9. And the results from Masson’s staining further revealed that SH-NPs inhibit the loss of collagen and improve its structural organization, enhancing the integrity of the dermal layer. This result is consistent with that of Yue et al. They reported that shikonin can inhibit MMP-9 overexpression and enhance blood-brain barrier permeability [52]. In summary, our results indicate that SH-NPs contribute to the maintenance of skin extracellular matrix homeostasis through regulation of MMP expression and collagen degradation.

Oxidative stress is triggered by an imbalance between the antioxidant and pro-oxidant systems in the body, characterized by elevated levels of ROS, diminished antioxidant enzymes, and subsequent cellular damage [53]. External toxins can induce oxidative stress in skin keratinocytes, exacerbating skin diseases such as psoriasis and atopic dermatitis [54]. According to studies by Hu et al., PM2.5 triggers oxidative stress in HaCaT cells, leading to oxidative damage and apoptosis [55]. This is consistent with findings that phenolic compounds from fruit wines significantly reduce MDA levels and enhance the activities of SOD, CAT, and GPx in rat synaptosomes under oxidative stress [56]. Piao et al. also demonstrated that exposure to PM2.5 increases intracellular ROS levels in HaCaT cells, subsequently inducing endoplasmic reticulum (ER) stress, mitochondrial damage, and autophagy [12]. These findings suggest that ROS play a common role in skin damage induced by PM2.5, as well as in other organs. Consistent with others, the results of this study also showed that PM2.5 exposure led to decreased SOD1/2 expression and increased MDA levels in mouse skin tissues. Furthermore, following treatment with SH-NPs (especially in the 2% dosage group), these oxygen stress indicators were restored, indicating that SH-NPs possess the ability to modulate oxidative stress. Research has demonstrated that shikonin protects the skin by enhancing antioxidant defenses in a UV irradiation model [57]. Altogether, these findings suggest that “inhibiting excessive ROS generation” is one of the core mechanisms through which SH-NPs protect against skin damage.

Inflammation is also recognized as one of the core pathological mechanisms underlying the development of most skin diseases. Kim et al. demonstrated that PM2.5 exposure activates the MAPK signaling pathway, leading to the upregulation of the inflammatory marker COX2 [58]. Vasarri et al. proved that excessive oxidative stress triggered by reactive oxygen species (ROS) can initiate inflammatory responses, leading to a vicious cycle in which oxidative stress and chronic inflammation mutually exacerbate each other [59]. Inflammation alone or together with ROS could degrade extracellular matrix components through activation of MMPs, leading to skin thinning and wrinkle formation [60]. The results of this study demonstrate that exposure to PM2.5 led to increased levels of inflammatory factors TNF-α, IL-1β, and NO in mouse skin tissues. However, after treatment with SH-NPs (especially in the 2% dose group), the levels of these inflammatory factors were significantly restored, indicating that SH-NPs possess considerable anti-inflammatory capacity. Other research showed that shikonin exerted anti-inflammatory ability by inhibiting the activation of the NLRP3 inflammasome and the NF-κB signaling pathway [61,62]. In conclusion, “inhibition of inflammatory response” may represent another core mechanism, in addition to “scavenging ROS”, by which SH-NPs protect against PM2.5-induced skin damage. Together, these two mechanisms, antioxidant ability and anti-inflammatory capacity, form a “dual defense line” through which SH-NPs intervene in skin damage induced by PM2.5 exposure. The SH-NPs can be further developed into specific anti-inflammatory and antioxidant repair products, bringing new possibilities for clinical and consumer markets.

The in vivo safety profile of SH-NPs establishes a solid foundation for their therapeutic applications. No significant histological alterations were observed in major organs, indicating that SH-NPs do not induce apparent tissue damage. Furthermore, the treated mice exhibited stable body weight and normal behavior. These findings suggest that SH-NPs are not only benign carriers but also highly promising therapeutic agents. They can alleviate PM2.5-induced skin damage without introducing additional safety concerns, thereby significantly enhancing their potential for clinical translation. The development of advanced delivery systems for shikonin to enhance its bioavailability and allow for the safe application of higher doses warrants further investigation in the future.

However, this study has several limitations. First, the composition of PM2.5 is complex, and its mechanism of inducing skin damage may vary depending on the source and composition. Although the use of standard samples in our present study ensures the reproducibility of experiments, it cannot replace experiments with samples from the real world. Therefore, future studies are needed to further evaluate the protective effects of SH-NPs against PM2.5 with different sources, especially from real-world-derived PM2.5. Second, although short-term experiments indicate that SH-NPs exhibit good biosafety, their long-term safety still requires validation through systematic toxicological studies. Additionally, this study did not directly examine the regulatory effects of SH-NPs on the potential signal pathways, such as NF-κB and MAPK signaling pathways. In further study, in vitro and in vivo experiments will be designed to validate the underlying mechanisms using Western blot and other assays. It is worth noting that the current particle size of SH-NPs is relatively large, which may limit their skin penetration efficiency, as studies have shown that smaller particles generally penetrate deeper into the skin more effectively. Therefore, optimizing the formulation’s particle size will be a key direction for future improvements in drug delivery systems. Finally, the metabolic pathways, pharmacokinetic behavior, and specific molecular mechanisms of SH-NPs remain to be elucidated.

## 5. Conclusions

In summary, this study successfully developed an efficient nanodelivery system for shikonin, termed SH-NPs, which effectively mitigates PM2.5-induced skin damage through multiple mechanisms, including the enhancement of antioxidant capacity, the suppression of inflammatory responses, and the maintenance of extracellular matrix homeostasis. These findings not only provide a novel therapeutic strategy for PM2.5-induced skin injury and provide a valuable technical framework for clinically applying other hydrophobic natural compounds. Future research will focus on evaluating the protective effects and underlying mechanisms of SH-NPs against skin injury induced by various environmental pollutants, and conducting further pre-clinical studies on safety and efficacy to facilitate clinical translation.

## Figures and Tables

**Table 1:** Primer sequences for mice genes.

Gene Name	Primer Sequence (5′–3′ Direction)	Genes Accession Numbers
*SOD1*	F: AACCAGTTGTGTTGTCAGGAC	NM_011434.2
	R: CCACCATGTTTCTTAGAGTGAGG	
*SOD2*	F: CAGACCTGCCTTACGACTATGG	NM_013671.3
	R: CTCGGTGGCGTTGAGATTGTT	
*TNF-α*	F: TAGCCCACGTCGTAGCAAAC	NM_013693.3
	R: TGTCTTTGAGATCCATGCCGT	
*IL-1β*	F: TGCCACCTTTTGACAGTGATG	NM_008361.4
	R: TGATGTGCTGCTGCGAGATT	
*Actin*	F: CGTTGACATCCGTAAAGACCTC	NM_007393.5
	R: TAGGAGCCAGGGCAGTAATCT	

## Data Availability

The datasets used or analyzed during the current study are available from the corresponding author upon reasonable request.

## Supplementary Materials

The following supporting information can be downloaded at: https://www.mdpi.com/article/10.3390/antiox14111301/s1. The flowchart illustrating the construction of SH-NPs is provided in Figure S1 in the attachment.
