# Supplementary figures and images for: Shikonin-Loaded Nanoparticles Attenuate Particulate Matter-Induced Skin Injury by Inhibiting Oxidative Stress and Inflammation
Source: Antioxidants (Basel). 2025 Oct 29;14(11):1301. doi: 10.3390/antiox14111301 (PMC12649448; doi:10.3390/antiox14111301)

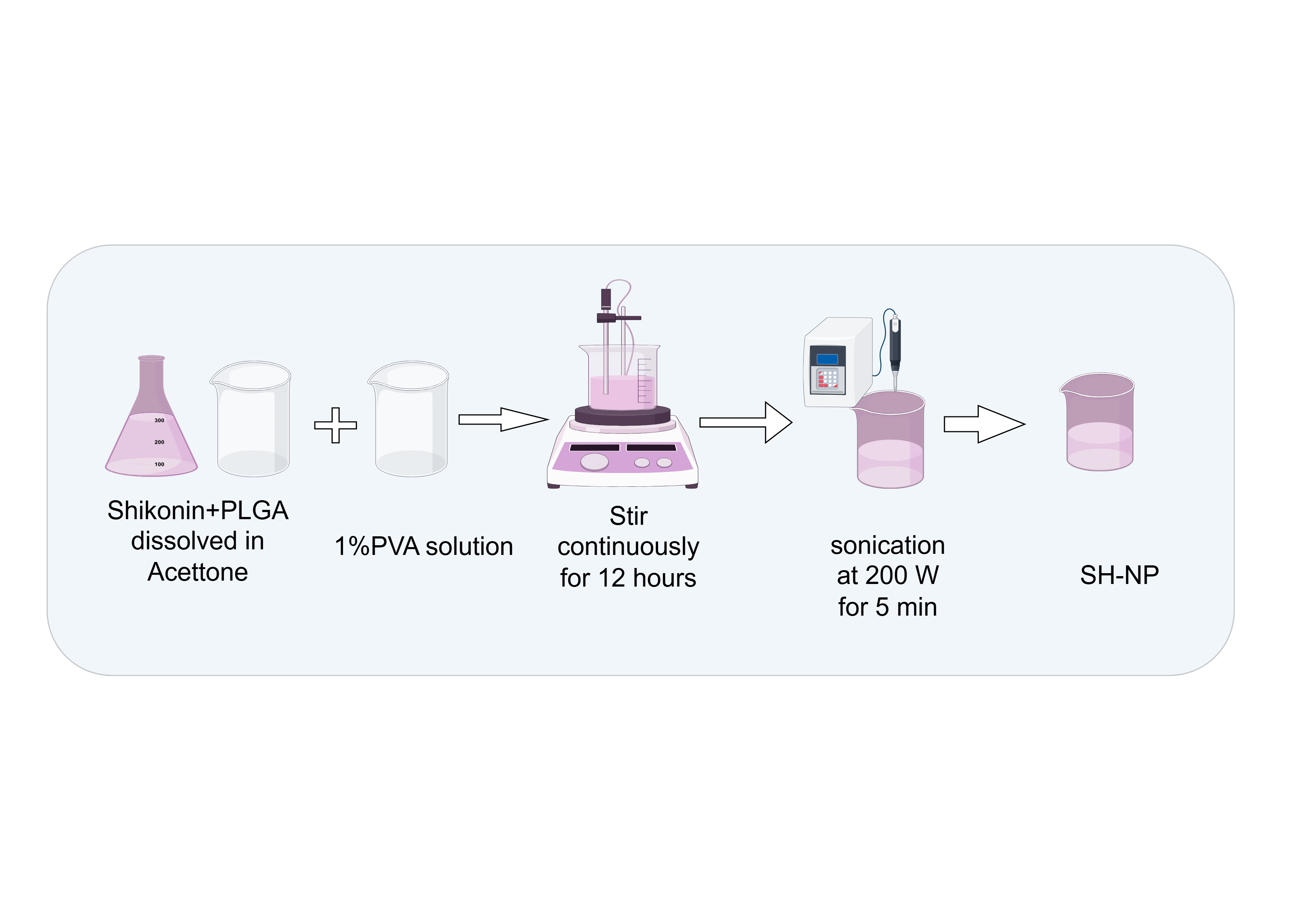

Supplement: Supplementary file 1 [file antioxidants-14-01301-s001.zip › antioxidants-3906281-supplementary.png]
